# Giant unruptured sinus of Valsalva aneurysms causing angina pectoris

**DOI:** 10.1186/s13019-024-02730-x

**Published:** 2024-04-20

**Authors:** Peng Hu, Zhedong Wan, Qianhui Sun, Peng Teng, Liang Ma

**Affiliations:** 1 Department of Cardiovascular Surgery, The First Affiliated Hospital, College of Medicine, Zhejiang University, #79 Qingchun Road, Hangzhou, 310003 Zhejiang Province P.R. China; 2 Department of Surgical Intensive Care Unit, The First Affiliated Hospital, College of Medicine, Zhejiang University, Hangzhou, P.R. China

**Keywords:** Sinus of Valsalva aneurysm, Left coronary sinus, Myocardial ischemia, Angina pectoris

## Abstract

**Background:**

Sinus of Valsalva aneurysm (SVA) is a rare cardiac anomaly which can be congenital or acquired. Patients with SVA are commonly asymptomatic when the occupying effect of SVA is insignificant, while ruptured SVA usually causes severe symptoms including heart failure and myocardial ischemia.

**Case presentation:**

We present an unusual case of a 64-year-old female manifesting with exertional dyspnea as well as angina pectoris for three months. Echocardiography and cardiac computed tomographic angiography confirmed unruptured left-coronary and non-coronary SVAs. The left anterior descending artery and left circumflex artery were stretched and compressed by the SVA which causing myocardial ischemia. The patient finally received aortic root replacement (Bentall procedure) and got symptom relieved.

**Conclusions:**

Giant unruptured SVA originating from left coronary sinus is extremely rare. Our case highlights that giant SVA should be considered in cases with angina pectoris. Echocardiography and coronary computed tomographic angiography are useful and important for diagnosis. Surgery is highly recommended in patients with SVA.

**Supplementary Information:**

The online version contains supplementary material available at 10.1186/s13019-024-02730-x.

## Introduction

Sinus of Valsalva aneurysm (SVA) is a rare cardiac anomaly with an estimated prevalence of 0.09% in the general population based on autopsy findings [1]. It is defined as enlargement of the aortic root area between the aortic valve annulus and the sinotubular junction. SVA can be congenital or acquired, which accounting for about 3.5% of the total congenital heart defects [2]. It is worth noting that SVA shows obvious racial and gender differences as Asian populations are five times more likely to be affected than Western [3] and males are two to four times more likely to be affected than females [4]. SVAs most commonly affect right-coronary sinus, followed by the noncoronary sinus, while left-coronary sinus SVA only accounts for less than 5% cases [5]. Ruptured SVA often causes dyspnea, chest pain, heart failure or sudden cardiac death, while most unruptured SVAs are asymptomatic [2]. Once an unruptured SVA is diagnosed, surgery is highly recommended as there is little information about the natural history of unruptured SVA and the mean survival of unrepaired ruptured SVA is 3.9 years [6]. Herein, we reported an extremely rare case of unruptured left-coronary and non-coronary SVAs that extrinsically compressed left coronary artery and caused dyspnea and angina pectoris.

## Case presentation

**Fig. 1:**
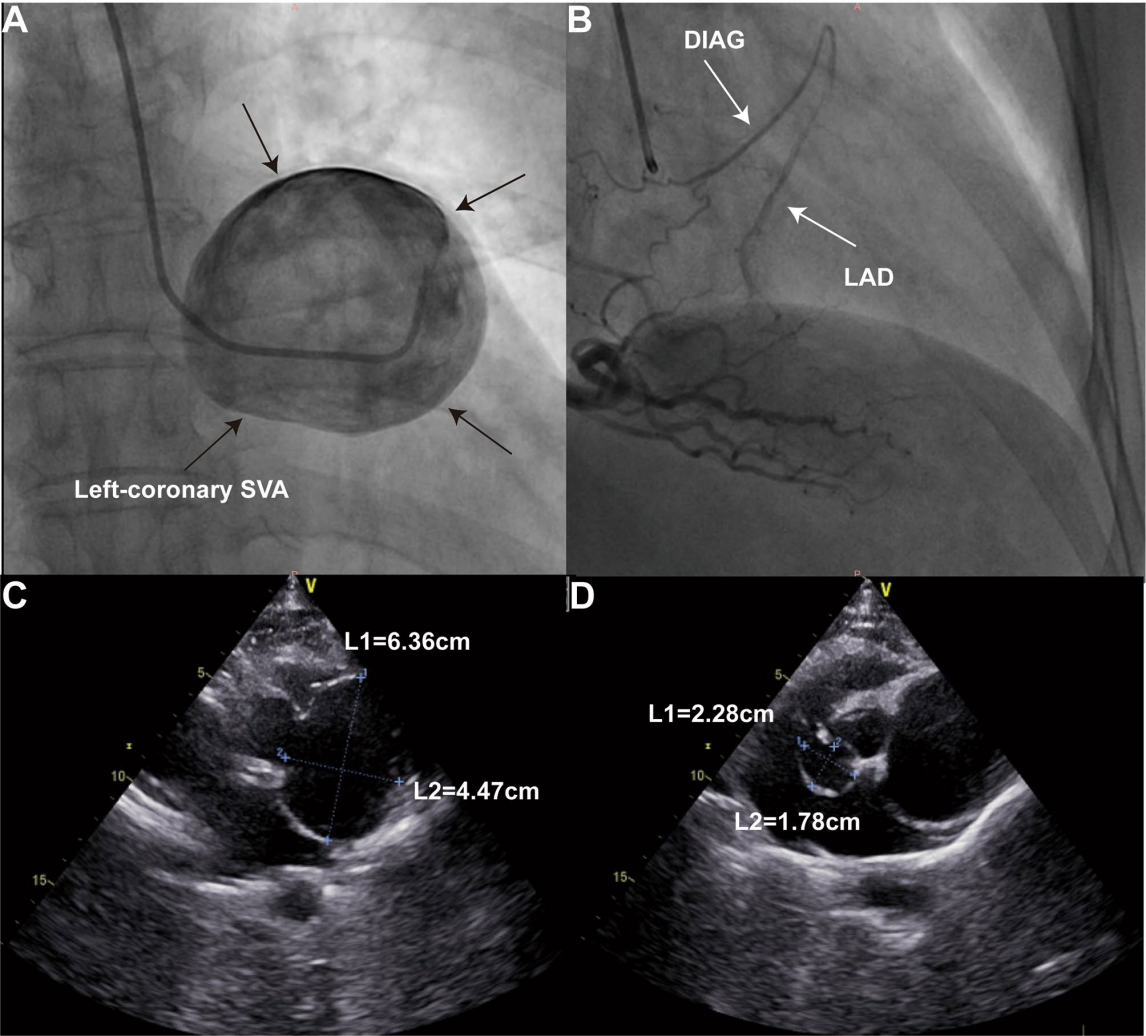
**(A)** Coronary angiography showed persist swirling of contrast within a large aneurysmal left-coronary sinus of Valsalva. **(B)** Left anterior descending artery was retrogradely perfused by the right coronary artery. Transthoracic echocardiography showed unruptured left-coronary **(C)** and non-coronary **(D)** sinus of Valsalva aneurysms. **SVA**: sinus of Valsalva aneurysm; **DIAG**: diagonal branch artery; **LAD**: left anterior descending artery

**Fig. 2:**
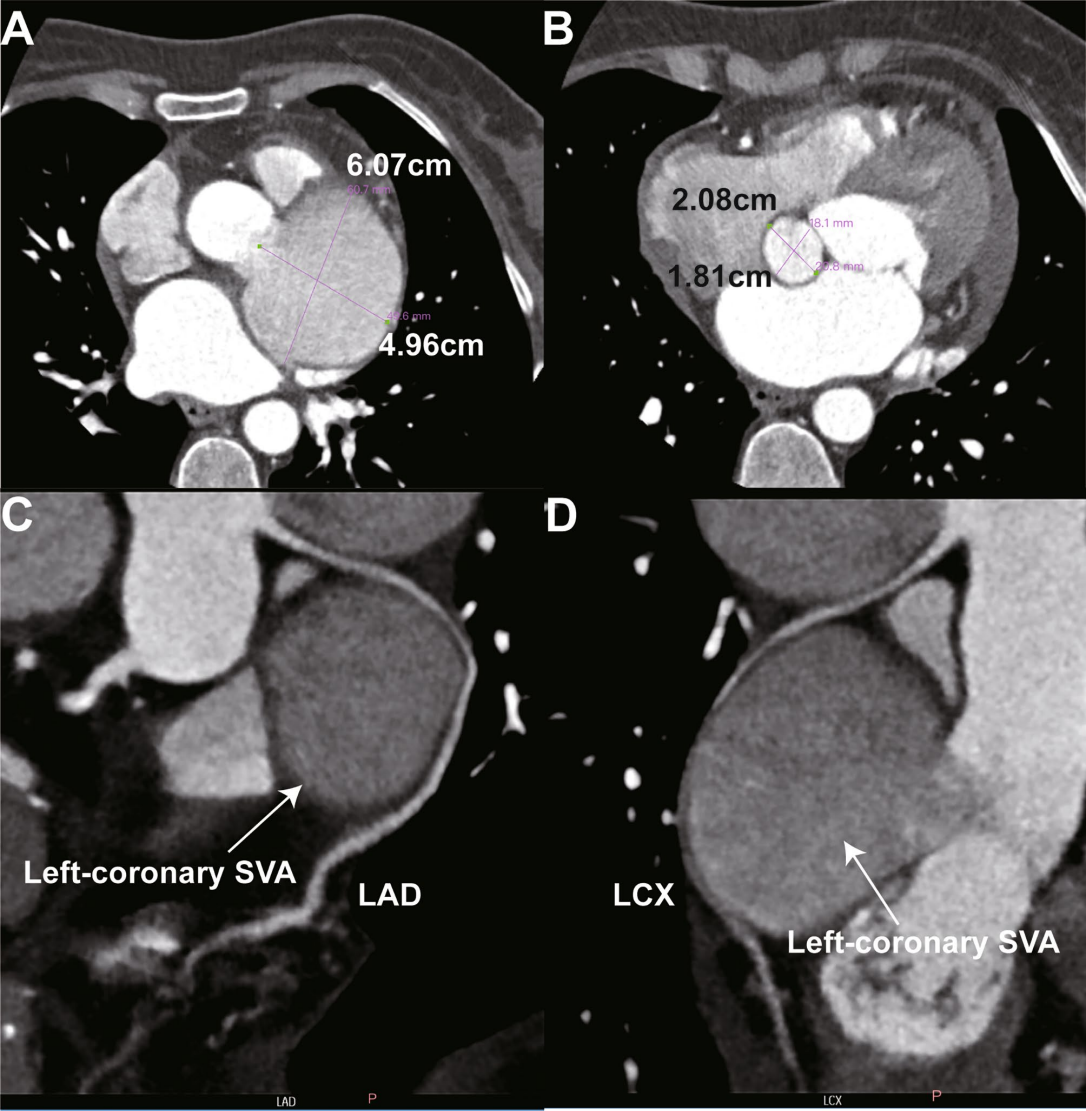
Coronary computed tomographic angiography showed unruptured left-coronary **(A)** and non-coronary **(B)** sinus of Valsalva aneurysms. The left anterior descending artery **(C)** and left circumflex artery **(D)** were stretched and compressed by the left-coronary sinus of Valsalva aneurysm. **SVA**: sinus of Valsalva aneurysm; **LAD**: left anterior descending artery; LCX: left circumflex artery

**Fig. 3:**
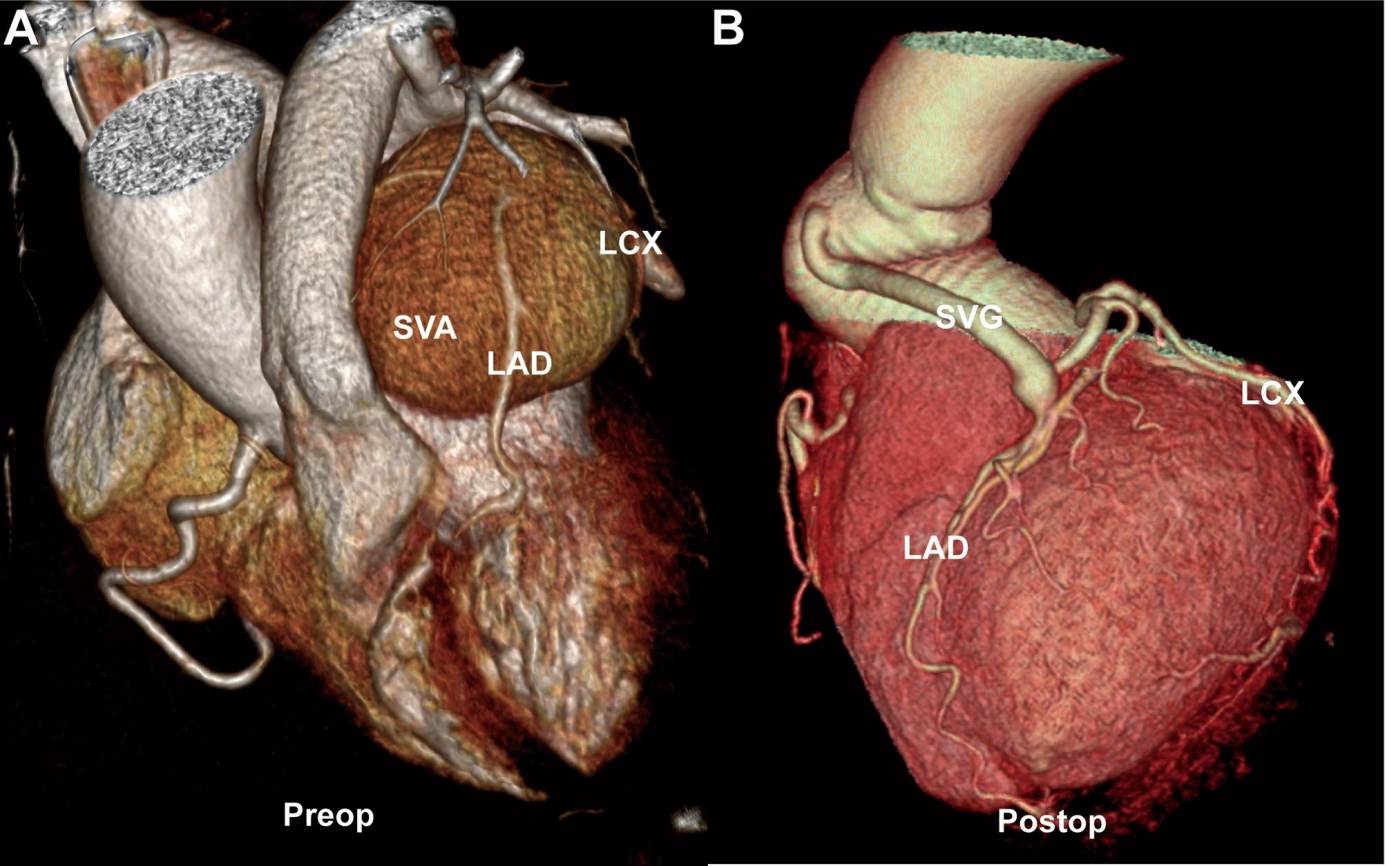
**(A)** Preoperative three-dimensional reconstruction of the coronary computed tomographic angiography showed giant unruptured left-coronary sinus of Valsalva aneurysm and the compressed left anterior descending artery and left circumflex artery. **(B)** Postoperative three-dimensional reconstruction of the coronary computed tomographic angiography showed normal morphology of aortic root and patent vein graft without SVA recurrence. **SVA**: sinus of Valsalva aneurysm; **LAD**: left anterior descending artery; LCX: left circumflex artery; **SVG**: saphenous vein graft

A 64-year-old female was admitted with progressive exertional dyspnea and angina pectoris for six months. No relevant medical history, including smoking, trauma, tumor or surgery, has been reported. The electrocardiogram revealed sinus rhythm with ST-segment non-significant change. The myoglobin, creatine kinase-MB and troponin I were within normal range while N-terminal pro-brain natriuretic peptide levels was 2133 pg/ml. Coronary angiography (CAG) (Supplementary Video 1) showed persist swirling of contrast within a large aneurysmal left-coronary sinus of Valsalva (Fig. 1A) and left anterior descending artery was retrogradely perfused by the right coronary artery (Fig. 1B). The stenosis of left coronary artery cannot be determined during CAG. Further echocardiography and coronary computed tomographic angiography (CCTA) were performed. Transthoracic echocardiography showed both left-coronary and non-coronary SVAs. The left-coronary SVAs was giant (6.4 × 4.5 cm in diameter) and was close to the left atrium and main pulmonary artery (Fig. 1C). The non-coronary SVA was 2.8 × 1.8 cm in diameter (Fig. 1D). No intracardiac shunting or obvious wall motion abnormality was observed. The left ventricular ejection fraction was 54%. CCTA showed a giant left-coronary SVA about 6 × 5 cm in diameter (Fig. 2A) as well as a non-coronary SVA about 2 × 1.8 cm in diameter (Fig. 2B). The contrast media filling in left-coronary sinus was significantly damaged which suggested blood flow stasis (Fig. 1A). The left coronary artery was originated above the SVA and the right coronary artery was originated from right-coronary sinus of Valsalva (Fig. 3A). The left anterior descending artery (LAD) and left circumflex artery (LCX) were stretched and compressed by the SVA (Fig. 2C&D).

Cardiac surgery was performed by using standard cardiopulmonary bypass with ascending aorta and bicaval cannulation via median sternotomy. Intraoperative findings confirmed the diagnosis of giant unruptured left-coronary SVA. LAD and LCX were severely displaced and compressed by the SVA. Because the entire left sinus of Valsalva was severely dilated and no clearly boundary of its neck could be defined, SVA closure with bovine epicardial patch plus left coronary artery bypass were considered difficult. The Bentall procedure was finally performed using a handmade composite graft of 23 mm Medtronic Hancock II bioprosthetic valve and 28 mm Terumo artificial vessel. Moreover, the orifice of left coronary artery was closed by ligation and continuous suture, and then the proximal LAD was bypassed by using a great saphenous vein. The LCX could also be perfused by the retrograde blood flow from the great saphenous vein graft and was not bypassed concomitantly. The operation went smoothly and the heart re-beat automatically. The early postoperatively course was uneventful as the patient spent two days in cardiac care unit and recovered to daily activity on postoperative day (POD) 7. However, the patient developed deep sternal wound infection on POD 10. Simple debridement was useless, and the patient received Gao’s procedure (pectoralis major muscle turnover flap reconstruction) on POD 17 and finally discharged home on POD 33 with good wound healing. The patient was followed up for 5 years. The CCTA at postoperative year 5 showed normal morphology of aortic root and patent vein graft without SVA recurrence (Fig. 3B).

## Discussion

SVA is a rare cardiac anomaly with an estimated prevalence of 0.09% in the general population [1]. Moreover, its incidence is higher among Asians (0.46-3.57%) [3] as compared with Western surgical series (0.14-0.37%) [7]. SVA could be congenital or acquired. Congenital SVA is caused due to failure fusion between aortic media and annulus fibrous of aortic valve [6], and acquired form is associated with weakness of the elastic lamina, which is commonly seen in connective tissue disorders, syphilis, infective endocarditis, atherosclerosis and trauma [8]. Majority of SVAs originate from the right-coronary sinus (65-86%), followed by the non-coronary sinus (10-30%) while the left-coronary sinus is the least affected (<5%) [5]. However, cases of multiple SVAs are extremely rare [9, 10].

Un-ruptured SVAs are usually asymptomatic, unless the SVA is large enough to cause occupying effect to adjacent structures. SVAs can rupture into any cardiac chambers or spaces, more commonly into the low-pressure chambers like right atrium, right ventricle or pulmonary artery. Right-coronary and non-coronary SVAs tend to rupture into right sided heart chambers in majority cases (>90%) while left-coronary SVAs may rupture into any chambers or spaces [7]. Almost all the ruptured SVAs can cause symptoms due to intra-cardiac shunting or pericardial tamponade, including decreased exertional tolerance, heart failure, myocardial ischemia and even sudden cardiac death [11]. Further, several reports described that giant SVAs compressed the coronary arteries and resulted in angina pectoris [4, 10, 12]. Another reason that SVAs might cause myocardial ischemia is that swirling blood in the SVA results in thrombosis and compromised coronary blood flow [13]. In our case, the compression to the LAD and LCX caused by SVA, as well as swirling blood in the SVA, both inducing the compromised coronary blood flow, which caused the patient’s symptom of decreased exertional tolerance and angina pectoris. To the best of our knowledge, such multiple SVAs with giant unruptured left-coronary SVA causing myocardial ischemia and angina pectoris has never been reported before.

Echocardiography, CCTA, cardiac magnetic resonance imaging are useful for the diagnosis of SVAs. Moreover, three-dimensional reconstruction of the images provides much more accurate evaluation and comprehensive assessment of SVAs and their relationship with adjacent structures like coronary arteries and cardiac chambers. CAG is of limited value in patients with SVAs as the corresponding orifice of coronary artery are difficult to engage and the enlarged and weakened wall of aortic root is much more vulnerable to be injured.

Surgical repair is highly recommended once a SVA is diagnosed [14]. The surgical strategy depends on the origin, size, shunting, associated lesions of the SVA. The mainstream surgical strategies include aneurysm resection with/without concomitant coronary bypass, bovine epicardial patch closure, Bentall procedure, aneurysm closure with concomitant aortic valve replacement, transcatheter closure [2, 15, 16]. In our case, the SVAs were thought difficult to be closed or resected as the structure of aortic root was severely changed, so the Bentall procedure was finally performed. Moreover, the orifice of the left coronary artery could not be reimplanted into the root of the artificial vessel due to the huge anastomosis tension. As a result, orifice closure with coronary artery bypass was performed as well.

## Conclusions

Angina pectoris caused by unruptured giant left-coronary SVA is an extremely rare cardiac condition which has seldomly been aware. Our case suggests the possibility that unruptured SVA could cause severe symptoms, as well as that SVA should be considered in patients with myocardial ischemia. CCTA is of great valve in diagnosis and once a SVA identified, surgery is highly recommended.

### Electronic supplementary material

Below is the link to the electronic supplementary material.

Supplementary Material 1

## Data Availability

No datasets were generated or analysed during the current study.

## Abbreviations

CAG: Coronary angiography
CCTA: Coronary computed tomographic angiography
LAD: Left anterior descending artery
LCX: Left circumflex artery
POD: Postoperative day
SVA: Sinus of Valsalva aneurysm
